# Timing of cardiac resynchronization therapy device implantation in heart failure patients and its association with outcomes

**DOI:** 10.1002/clc.23135

**Published:** 2018-12-26

**Authors:** Sarah A. Goldstein, Robert J. Mentz, Anne S. Hellkamp, Tiffany C. Randolph, Gregg C. Fonarow, Adrian Hernandez, Clyde W. Yancy, Sana M. Al‐Khatib

**Affiliations:** ^1^ Division of Cardiology Duke University Hospital Durham North Carolina; ^2^ Duke Clinical Research Institute Durham North Carolina; ^3^ Division of Cardiology Ronald Reagan UCLA Medical Center, Ahmanson‐UCLA Cardiomyopathy Center Los Angeles California; ^4^ Division of Cardiology Northwestern University Medical Center Chicago Illinois

**Keywords:** cardiac resynchronization therapy, CRT, heart failure, mortality, outcomes, re‐hospitalization

## Abstract

**Background:**

When used in appropriately selected heart failure (HF) patients, cardiac resynchronization therapy (CRT) reduces mortality and hospitalization. It is not understood whether CRT implantation during hospitalization for HF is associated with similar benefits.

**Hypothesis:**

Timing of CRT implantation relative to hospitalization for HF is associated with clinical outcomes.

**Methods:**

This analysis included patients eligible for CRT and discharged alive between January 2005 and December 2012 from 388 hospitals in Get With The Guidelines‐HF. Participants were linked with Centers for Medicare and Medicaid Services data to evaluate outcomes of all‐cause mortality and HF re‐hospitalization based on CRT status (present on admission, placed during hospitalization, and prescribed at discharge; reference = no CRT).

**Results:**

Of 15 619 CRT‐eligible HF patients, 2408 (15%) had CRT on admission, 1269 (8%) underwent CRT implantation during hospitalization and 643 (4%) had CRT prescribed at discharge. Compared with patients without CRT, mortality was lower in those who received CRT implantation during HF hospitalization (adjusted hazard ratio [HR] 0.63; *P* < 0.0001) and those prescribed CRT at discharge (adjusted HR 0.78; *P* = 0.048). A reduction in HF re‐hospitalization was observed in patients with CRT implanted during hospitalization (adjusted HR 0.64; *P* < 0.0001), but not in those who were prescribed CRT at discharge (adjusted HR 1.02; *P* = 0.77).

**Conclusion:**

CRT implantation during HF hospitalization was associated with lower rates of mortality and HF re‐hospitalization. These data suggest that a CRT utilization strategy that does not delay implantation to the post‐discharge period may be appropriate. Randomized data are needed to definitively identify optimal timing of CRT implantation.

**Abbreviations**
CI: confidence interval
CMS: Centers for Medicare and Medicaid Services
CRT: cardiac resynchronization therapy
GWTG: get with the guidelines
HF: heart failure
HR: hazard ratio
LVEF: left ventricular ejection fraction
ms: milliseconds

## 1 INTRODUCTION

Heart failure (HF) currently affects an estimated 6.5 million adults in the United States, with prevalence continuing to rise.^1^, ^2^ When used in appropriately selected HF patients, cardiac resynchronization therapy (CRT) is associated with improved left ventricular function, lower mortality and HF hospitalizations, and improved quality of life.^3^, ^4^, ^5^, ^6^ CRT implantation is recommended in current national guidelines (Class I) for patients with symptomatic HF, left ventricular ejection fraction (LVEF) ≤ 35%, sinus rhythm, and left bundle branch block with QRS duration ≥150 ms with Class IIA recommendations for those with left bundle branch block and QRS duration 120 to 149 ms or with non‐left bundle branch block and QRS ≥ 150 ms.^7^, ^8^, ^9^ Landmark trials showing the benefit of CRT do not specify timing of implantation relative to hospitalization for HF. 4, 10 It is therefore not well understood whether CRT implantation during hospitalization for HF is associated with similar improvements in clinical outcomes compared with CRT implantation in an elective setting. CRT implantation during an HR hospitalization could provide benefit by targeting patients at highest risk for adverse outcomes after discharge.^11^ Alternatively, patients hospitalized for HF are subject to appreciable clinical instability and medication adjustment, which could attenuate the potential benefit of immediate CRT implantation. This analysis examines whether the timing of CRT implantation relative to admission for HF is associated with differences in all‐cause mortality and HF re‐hospitalization.

## 2 METHODS

### 2.1 Data sources

Data for this analysis were obtained from the get with the guidelines‐heart failure (GWTG‐HF) database as well as the Centers for Medicare and Medicaid Services (CMS) fee‐for‐service inpatient claims. The GWTG‐HF database has previously been described.^12^, ^13^, ^14^ Briefly, GWTG is a quality improvement program that collects clinical information from participating hospitals regarding the in‐hospital care and outcomes of consecutive patients hospitalized for coronary artery disease, stroke or HF. Data quality is monitored through frequent reports that review the completeness and accuracy of submitted data. Only sites with a high degree of completeness, defined as having medical history data for at least 75% of their patients, were included in this analysis. Individual patients with missing data for a key variable (age, sex, race, ejection fraction, and medical history) were also excluded. Prior reports have demonstrated that patient characteristics and health outcomes of Medicare beneficiaries with HF who are enrolled in the GWTG‐HF registry are similar to Medicare beneficiaries in the United States who are hospitalized for HF but are not enrolled.^15^, ^16^ All participating institutions are required to comply with local regulatory and privacy guidelines and to submit the GWTG‐HF protocol for review and approval by their institutional review board. Data are primarily used at the local site for quality improvement initiatives, and were therefore granted a waiver of informed consent under the common rule.

At each participating site, personnel are trained to collect data using standard definitions for demographical and clinical characteristics, previous treatments, contra‐indications to evidence‐based therapies and in‐hospital clinical outcomes. Data collection regarding CRT includes prior implantation, new implantation during hospitalization for HF, implantation prescribed at HF hospitalization discharge, and contraindications to CRT defibrillator or pacemaker. Reporting on QRS morphology and duration became required in 2011. The Duke Clinical Research Institute is the primary analytic center for the combined data. This study was approved by the Duke University Institutional Review Board.

### 2.2 Study population

We identified 47 357 GWTG‐HF patients with an LVEF ≤35% who required hospitalization for a primary diagnosis of HF, discharged alive between January 2005 and December 2012, and could be linked to Medicare data. Patients with a physician‐documented contraindication to CRT (including a narrow QRS duration, lack of optimal medical therapy for treatment of HF, acute myocardial infarction in the preceding 40 days, recent coronary revascularization, not NYHA functional class III or ambulatory class IV, and new onset HF) were excluded. Patients who were not discharged home, were discharged to comfort care or had missing documentation were also excluded. Finally, for patients with multiple admissions, only the first was analyzed. A final cohort of 15 619 patients was identified.

### 2.3 Outcomes

Outcomes of interest were all‐cause mortality and HF re‐hospitalization, defined as a hospital admission with any of the following primary ICD‐9‐CM diagnosis codes: 428.x, 402.x1, 404.x1, and 404.x3. Patients who were not reported to have died were censored on December 31, 2012 or the date at which they were no longer enrolled in Part A Fee‐for‐Service Medicare, whichever occurred first.

### 2.4 Statistical analysis

For all analyses, patients were grouped by timing of CRT implantation: prior to HF admission and during hospitalization and as CRT prescribed at discharge, or no CRT. Baseline characteristics of the 4 CRT groups are summarized as medians (25th and 75th percentiles) for continuous variables and as percentages (frequencies) for categorical variables. Mortality rates are summarized using Kaplan‐Meier rates.

To compare mortality risk between groups, Cox proportional hazard models were used. Cumulative incidence rates and Fine and Gray models, which consider death as a competing risk, were used to compare hospital re‐hospitalization rates between groups.

The interaction between time and CRT group was tested in each model, and all models were adjusted; covariates are listed at Table 2. Covariates were chosen prior to analysis to best account for differences between groups that may have affected the outcomes of interest and represent those used in other GWTG analyses. Multiple imputations (25 iterations) was used to account for missing covariate values in Cox models. Because QRS duration reporting was not required prior to 2011 leading to missing QRS data, a sensitivity analysis was performed that included only admissions in 2010 to 2012 and confined the no‐CRT comparator group to those with documented QRS ≥ 120 ms.

A *P*‐value of <0.05 was considered statistically significant for all tests. Analyses were performed using SAS version 9.4 or higher (SAS Institute, Cary, North Carolina).

## 3 RESULTS

### 3.1 Study population

Around half of all participating sites were excluded due to a low degree of data completeness. Fully participating sites were similar to omitted sites in geographic region, urban vs rural location, teaching status, and size. There were no patients excluded for key missing data. Overall, the rate of missing data for individual variables was low, with a missing rate of <15% for all variables except BMI (26%).

### 3.2 Baseline characteristics

Among the 15 619 patients from 388 sites who met the inclusion criteria, 4320 (28%) received CRT; 2408 (15%) had CRT in place at the time of admission, 1269 (8%) underwent CRT placement during index hospitalization, and 643 (4%) had CRT prescribed at the time of discharge (Table 1). Most patients received or were prescribed CRT‐D, with only 7% receiving CRT‐P. Baseline characteristics of the study population are shown in Table 1. Across all groups, most patients were men, white, and had ischemic heart disease. Compared with other groups, patients with CRT in place at admission had higher rates of prior atrial arrhythmia and ischemic heart disease. The same group also had higher rates of non‐cardiovascular comorbidities including anemia, chronic renal insufficiency, diabetes, depression, and history of cerebrovascular accident or transient ischemic attack. Rates of guideline‐recommended medical therapy were high in all groups.

**Table 1:** Baseline characteristics for patients by CRT implant group^a^

Baseline characteristic	CRT in place at admission	CRT implanted in hospital	CRT prescribed at discharge	No CRT
Total patients	2408	1269	643	11 299
Age (years)	77 (72, 83)	76 (71, 81)	77 (71, 82)	78 (71, 84)
Female	28% (682)	28% (357)	33% (215)	40% (4520)
Race				
White	83% (2007)	88% (1115)	81% (520)	78% (8816)
Black	11% (260)	7% (90)	10% (65)	14% (1566)
Other	6% (141)	5% (64)	9% (58)	8% (917)
Presentation				
Systolic blood pressure	125 (110, 144)	129 (114, 144)	133 (117, 149)	134 (117, 153)
Heart rate	77 (70, 88)	74 (64, 84)	80 (70, 92)	84 (72, 98)
LVEF, %	25 (20, 30)	25 (20, 30)	25 (20, 30)	25 (20, 30)
Body mass index	25.9 (22.8, 29.7)	26.6 (23.5, 30.5)	26.5 (22.8, 30.9)	25.7 (22.2, 29.7)
Medical history				
Anemia	16% (387)	10% (129)	14% (89)	14% (1574)
Prior atrial arrhythmia	48% (1166)	40% (503)	44% (280)	33% (3698)
Prior CVA or TIA	15% (372)	12% (155)	12% (78)	14% (1556)
Dialysis	2% (56)	1% (15)	3% (16)	3% (323)
Chronic renal insufficiency	24% (580)	13% (162)	21% (137)	19% (2182)
Depression	9% (215)	6% (76)	5% (30)	7% (807)
Diabetes	42% (1010)	35% (448)	40% (257)	39% (4422)
Insulin treated	18% (442)	15% (194)	16% (101)	15% (1729)
Non‐insulin treated	24% (577)	20% (258)	25% (160)	24% (2724)
Hyperlipidemia	53% (1277)	58% (738)	54% (347)	47% (5349)
Hypertension	69% (1666)	69% (869)	69% (441)	73% (8190)
Ischemic heart disease	78% (1885)	71% (905)	73% (469)	66% (7491)
Prior PCI or CABG	54% (1301)	49% (627)	48% (310)	39% (4440)
Peripheral arterial disease	15% (363)	14% (172)	12% (77)	13% (1501)
COPD # or asthma	28% (663)	23% (294)	25% (159)	27% (3008)
Smoking in past 12 months	9% (214)	11% (136)	11% (70)	13% (1447)
Medications				
ACE‐inhibitor # or ARB	90% (1585)	94% (999)	95% (458)	90% (8001)
Beta blocker	95% (2102)	96% (1150)	97% (571)	93% (9739)
Aldosterone antagonist	34% (691)	32% (373)	38% (207)	24% (2444)
Anticoagulant therapy	56% (1119)	45% (500)	56% (269)	40% (3644)
Hospital characteristics				
Geographic region				
Northeast	29% (707)	28% (360)	31% (197)	30% (3347)
Midwest	27% (642)	30% (383)	22% (143)	24% (2652)
South	34% (809)	30% (378)	35% (227)	35% (3902)
West	10% (250)	12% (148)	12% (76)	12% (1398)
Teaching hospital	65% (1566)	74% (941)	62% (396)	63% (7055)
Rural site	5% (113)	1% (10)	3% (18)	6% (727)
Number of beds	431 (283, 601)	475 (368, 616)	396 (269, 556)	396 (244, 559)
Able to perform heart transplants	19% (407)	23% (286)	15% (81)	12% (1252)

Abbreviations: ACE, angiotensin converting enzyme; ARB, angiotensin II receptor blocker; CABG, coronary artery bypass graft; CRT, cardiac resynchronization therapy; CVA, cerebrovascular accident; LVEF, left ventricular ejection fraction; PCI, percutaneous coronary intervention; TIA, transient ischemic attack.

a As this table is intended to describe the analysis cohort, rather than to test any hypotheses about the underlying population, significance tests were not performed.

### 3.3 Mortality and CRT use

**Figure 1:**
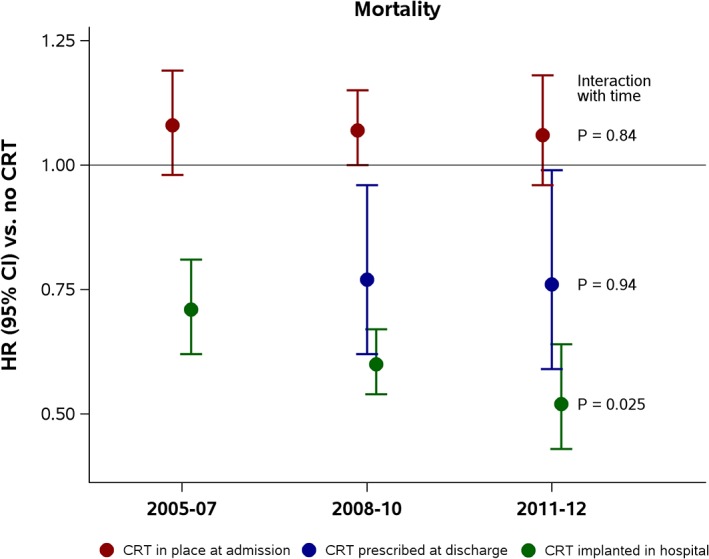
Relationships between time of CRT implantation and mortality**—**the risk of death is lower for CRT implantation during hospitalization for HF vs CRT in place at admission or CRT prescription at discharge. These relationships remain consistent over time.CRT, cardiac resynchronizatio therapy; HF, heart failure; HR, hazard ratio; CI, confidence interval

Table 2 demonstrates mortality by CRT implantation group expressed as Kaplan‐Meier event rates, both for the overall study period and by time period. Patients who received CRT during the index admission had a lower risk of death compared with patients who had no CRT (adjusted HR 0.63, 95% confidence interval [CI] 0.57, 0.70; *P* < 0.0001). This associated benefit increased over time (adjusted HR 0.7‐0.52; *P* = 0.025). Patients with CRT in place at the time of HF admission had a higher risk of mortality compared with patients who had no CRT (adjusted HR 1.08, 95% CI 1.01, 1.15; *P* = 0.034). This finding remained consistent over time (*P* = 0.84). Patients who were prescribed CRT at discharge also had lower risk of death compared with those who did not receive CRT (HR 0.78, 95% CI 0.65, 0.93; *P* = 0.0048). This associated risk reduction remained consistent over time (*P* = 0.094) and was not as large as that seen in those with CRT implanted during admission. These relationships are demonstrated graphically in Figure 1. A sensitivity analysis confining the no‐CRT comparator group to only those with documented prolonged QRS complexes revealed similar hazard ratios (Supporting Information Table S1).

**Table 2:** Mortality by CRT implant group and time period^a^

	All‐cause mortality stratified by time period of heart failure hospitalization			
	All time periods	2005‐07	2008‐10	2011‐12
CRT in place at admission				
Total patients	2408	826	844	738
Total events	1519	687	593	239
Event rate at 1 year (95% CI)	35.0% (33.0, 37.0)	34.5% (31.3, 37.8)	33.2% (30.2, 36.6)	38.8% (34.6, 43.3)
Event rate at 3 years (95% CI)	65.2% (63.0, 67.4)	64.8% (61.5, 68.1)	64.5% (61.1, 67.8)	NA
*P* for CRT × time interaction	0.84	—	—	—
HR (95% CI) CRT vs no CRT	1.08 (1.01, 1.15)	1.08 (0.98, 1.19)	1.07 (1.00, 1.15)	1.06 (0.96, 1.18)
*P*	0.034	0.12	0.043	0.26
CRT implanted during admission				
Total patients	1269	321	624	324
Total events	522	208	244	70
Event rate at 1 year	15.7% (13.7, 17.8)	13.0% (9.7, 17.2)	15.5% (12.9, 18.6)	18.9% (14.7, 24.2)
Event rate at 3 years	38.5% (35.5, 41.7)	36.2% (31.1, 41.9)	37.6% (33.7, 41.8)	NA
*P* for CRT × time interaction	0.025	—	—	—
HR (95% CI) CRT vs no CRT	0.63 (0.57, 0.70)	0.71 (0.62, 0.81)	0.60 (0.54, 0.67)	0.52 (0.43, 0.64)
*P*	<0.0001	<0.0001	<0.0001	<0.0001
CRT prescribed at discharge				
Total patients	643	—	266	377
Total events	221	—	139	82
Event rate at 1 year	26.3% (22.7, 30.3)	—	26.9% (21.9, 32.6)	26.3% (21.2, 32.4)
Event rate at 3 years	49.7% (44.4, 55.3)	—	49.8% (43.8, 56.1)	NA
*P* for CRT × time interaction	0.94	—	—	—
HR (95% CI) CRT vs no CRT	0.78 (0.65, 0.93)	—	0.77 (0.62, 0.96)	0.76 (0.59, 0.99)
*P*	0.0048	—	0.021	0.041
No CRT				
Total patients	11 299	4382	3836	3081
Total events	6682	3442	2373	867
Event rate at 1 year	30.3% (29.5, 31.2)	28.5% (27.2, 29.9)	31.1% (29.7, 32.6)	32.8% (30.8, 34.9)
Event rate at 3 years	57.6% (56.6, 58.7)	55.4% (53.9, 56.9)	59.6% (58.0, 61.3)	NA

Abbreviations: CI, confidence interval; CRT, cardiac resynchronization therapy; HR, hazard ratio.

a Event rates are Kaplan‐Meier rates. All *P*‐values and hazard ratios are from adjusted Cox models. Follow‐up duration shown as median (IQR). Models contain the following covariates: age, gender, race, left ventricular ejection fraction, systolic blood pressure, heart rate, body mass index, medical history (anemia, prior atrial arrhythmia, prior cerebrovascular accident or transient ischemic attack, ischemic heart disease, pulmonary disease, depression, diabetes, hypertension, hyperlipidemia, peripheral artery disease, renal insufficiency, chronic dialysis, smoking, prior revascularization), medications at discharge (angiotensin converting enzyme‐inhibitor or angiotensin II receptor blocker, beta blocker, aldosterone antagonist, anticoagulant), and hospital characteristics (geographic region, rural location, teaching hospital, number of beds, and whether the hospital performs heart transplants). Missing values for covariates were imputed using multiple imputations (25 iterations).

### 3.4 HF re‐hospitalization and CRT use

**Figure 2:**
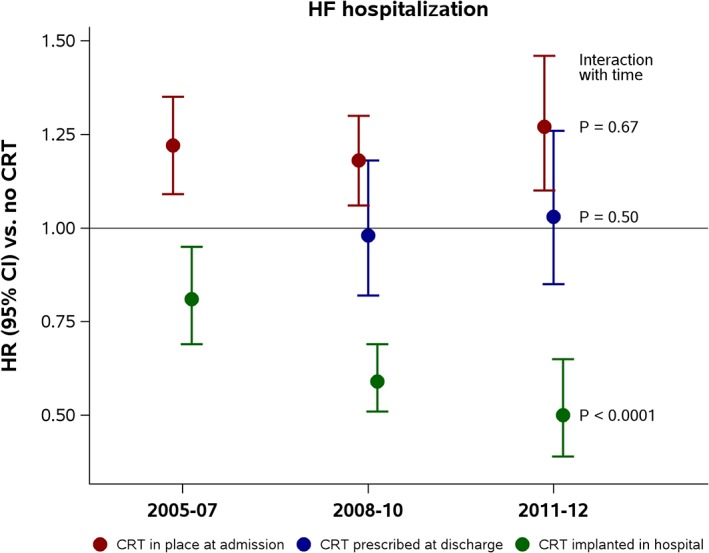
Relationships between time of CR implantation and HF re‐hospitalization—the risk of HF re‐hospitalization is lower for CRT implantation during hospitalization for HF vs CRT in place at admission or CRT prescription at discharge. These relationships remain consistent over time. CRT, cardiac resynchronization therapy; HF, heart failure; HR, hazard ratio; CI, confidence interval

Table 3 shows HF re‐hospitalization by CRT implantation group expressed as cumulative incidence rates, both for the overall study period and by time period. Patients who received CRT during admission had a lower associated risk of HF re‐hospitalization compared with patients who had no CRT (adjusted HR 0.64, 95% CI 0.58, 0.71; *P* < 0.0001). This association became more pronounced over time (adjusted HR of 0.81‐0.50; *P* < 0.0001). Patients who had CRT at the time of HF admission had a higher associated risk of HF re‐hospitalization compared with patients who had no CRT (adjusted HR 1.22, 95% CI 1.14, 1.30; *P* < 0.0001). This finding was consistent over time (*P* = 0.67). Patients with CRT prescribed at discharge had a risk of HF re‐hospitalization that was similar to those without CRT (HR 1.02, 95% CI 0.89, 1.16; *P* = 0.77), a finding that was consistent over time (*P* = 0.50). A graphical representation of these relationships is shown in Figure 2. A sensitivity analysis confining the no‐CRT comparator group to only those with documented prolonged QRS complexes revealed similar hazard ratios (Table S2).

**Table 3:** Heart failure hospitalization by CRT implant group and time period^a^

	Heart failure re‐hospitalization stratified by time period of heart failure hospitalization			
	All time periods	2005‐07	2008‐10	2011‐12
CRT in place at admission				
Total events	1282	488	501	293
Event rate at 1 year (95% CI)	45.3% (43.3,47.4)	43.8% (40.5,47.3)	46.1% (42.8,49.6)	46.0% (42.0,50.4)
Event rate at 3 years (95% CI)	57.0% (54.9,59.2)	55.1% (51.7,58.6)	58.6% (55.3,62.1)	NA
*P* for CRT × time interaction	0.67	—	—	—
HR (95% CI) CRT vs no CRT	1.22 (1.14, 1.30)	1.22 (1.09, 1.35)	1.18 (1.06, 1.30)	1.27 (1.10, 1.46)
*P*	<0.0001	0.0003	0.0021	0.0012
CRT implanted during admission				
Total events	419	149	207	63
Event rate at 1 year	21.9% (19.7,24.4)	24.4% (20.1,29.6)	22.1% (19.0,25.6)	18.6% (14.6,23.8)
Event rate at 3 years	34.0% (31.2,37.0)	38.2% (33.2,44.0)	32.8% (29.2,36.8)	NA
*P* for CRT × time interaction	<0.0001	—	—	—
HR (95% CI) CRT vs no CRT	0.64 (0.58, 0.71)	0.81 (0.69, 0.95)	0.59 (0.51, 0.69)	0.50 (0.39, 0.65)
*P*	<0.0001	0.0092	<0.0001	<0.0001
CRT prescribed at discharge				
Total events	251	—	133	116
Event rate at 1 year	37.6% (33.7,41.9)	—	38.5% (33.0,44.8)	36.9% (31.5,43.2)
Event rate at 3 years	48.9% (44.2,54.0)	—	49.6% (43.9,56.1)	NA
*P* for CRT × time interaction	0.50	—	—	—
HR (95% CI) CRT vs no CRT	1.02 (0.89, 1.16)	—	0.98 (0.82, 1.18)	1.03 (0.85, 1.26)
*P*	0.77	—	0.86	0.75
No CRT				
Total events	5201	2310	1906	987
Event rate at 1 year	37.1% (36.2,38.1)	36.0% (34.6,37.4)	38.8% (37.3,40.4)	36.5% (34.6,38.5)
Event rate at 3 years	48.8% (47.8,49.8)	48.2% (46.7,49.7)	49.8% (48.2,51.5)	NA

Abbreviations: CI, confidence interval; CRT, cardiac resynchronization therapy; HR, hazard ratio.

a Event rates are cumulative incidence rates. All *P*‐values and hazard ratios are from adjusted Fine and Gray models. Models contain the following covariates: age, gender, race, left ventricular ejection fraction, systolic blood pressure, heart rate, body mass index, medical history (anemia, prior atrial arrhythmia, prior cerebrovascular accident or transient ischemic attack, ischemic heart disease, pulmonary disease, depression, diabetes, hypertension, hyperlipidemia, peripheral artery disease, renal insufficiency, chronic dialysis, smoking, prior revascularization), medications at discharge (angiotensin converting enzyme‐inhibitor or angiotensin II receptor blocker, beta blocker, aldosterone antagonist, anticoagulant), and hospital characteristics (geographic region, rural location, teaching hospital, number of beds, and whether the hospital performs heart transplants). Missing values for covariates were imputed using multiple imputations (25 iterations).

## 4 DISCUSSION

This analysis utilizing the GWTG‐HF registry has several notable findings: (a) CRT implantation during HF hospitalization is associated with improvements in risk‐adjusted mortality and HF re‐hospitalization compared with no CRT, (b) CRT prescribed at discharge is associated with improved risk‐adjusted mortality compared with no CRT, but this effect is not as large as that seen in CRT implanted during HF hospitalization, (c) CRT prescribed at discharge is not associated with improved HF re‐hospitalization rates compared with no CRT, and (iv) pre‐existing CRT at the time of HF hospitalization is associated with worse clinical outcomes compared with no CRT.

Current guidelines do not address timing of CRT implantation relative to admission for HF. Our analysis shows that in eligible patients admitted with a primary diagnosis of HF, CRT implantation prior to discharge is associated with improved mortality and HF hospitalization rates compared to those who do not receive CRT. In current practice, it is uncommon for CRT to be implanted during HF hospitalization, a fact that is supported in our patient cohort, as only 8% of patients without CRT already in place received the therapy prior to discharge. These results suggest that it is appropriate to consider CRT implantation in patients admitted for HF prior to discharge.

The present study also shows that patients who are prescribed CRT at discharge with a plan for subsequent placement is associated with only modest clinical benefit compared to those who receive CRT during HF hospitalization. Although it is unknown whether patients in this group received post‐discharge CRT as prescribed, this represents real‐world practice making the results clinically applicable. Given the known clinical benefit of CRT, long‐term follow‐up to further investigate rates of CRT implantation when prescribed at discharge could highlight important areas for improvement in discharge transitions and planning. Additionally, prospective, randomized trials aimed at comparison of clinical outcomes between patients who receive CRT during admission vs after discharge could further our understanding of appropriate timing of device implantation.

In our analysis, patients who require hospitalization for HF with pre‐existing CRT have higher mortality and HF re‐hospitalization rates than other groups studied. Based on differences in baseline characteristics highlighted in Table 1, this group represents a patient cohort with more cardiovascular and non‐cardiovascular comorbidities. Despite adjusting for the majority of these differences, worsened clinical outcomes persisted. This suggests the presence of unmeasured clinical attributes, likely including markers of HF severity or duration of disease, which may account for the recurrent hospitalizations and worsened mortality. Prior studies and subsequent guidelines have focused on indications for CRT implantation, but have not identified markers of HF disease severity that may be associated with lack of clinical benefit or worsened outcomes with CRT use. Our results suggest that a better understanding of specific factors that may affect certain patients' ability to receive the intended clinical benefits of CRT is needed.

### 4.1 Strengths and limitations

The major strength of our analysis is that it utilized data collected as part of a large, national registry. Because of the large number of subjects included, the results are statistically robust with enhanced data consistency. In addition, registry analysis provides a diverse patient population not typically included in clinical trials.

A major limitation of our study is that patients were not randomized and thus our results are associations as opposed to causal relationships. As an observational study, measured or unmeasured confounding may have affected the findings of this analysis. To address this weakness, identified differences were adjusted for using robust statistical modeling. Physicians' perception of clinical stability or instability, a factor that likely affected the decision regarding timing of ICD implantation, was unknown and therefore could be accounted for. Another limitation inherent to a registry analysis is that some important data elements may not be captured. Because reporting on QRS morphology and duration were not required prior to 2011, QRS data were missing on many patients included in our analysis. We do not believe that lack of QRS data significantly impacted our analysis as we excluded patients determined not to qualify for CRT by the treating physician on the basis of a narrow QRS among other reasons. Furthermore, our results did not significantly differ in a sensitivity analysis that confined the no‐CRT comparator group to only those with documented prolonged QRS duration. Because only sites that submitted medical history data for at least 75% of their patients were included, about half of the sites that participate in the GWTG‐HF registry were excluded. Fully participating sites, however, were similar to omitted sites in geographic region, urban vs rural location, teaching status and size. Therefore, it is reasonable to assume that the patients included represent an unbiased sample from the larger population.

## 5 CONCLUSION

Landmark trials and current clinical practice guidelines do not address optimal timing of CRT implantation relative to hospitalization for HF. The primary finding of this analysis is that CRT implantation during HF hospitalization is associated with lower all‐cause mortality and reduced HF re‐hospitalization rates compared with patients who do not receive CRT. Clinical benefit is greater when CRT is implanted during HF hospitalization compared with when it is prescribed at discharge. These data suggest that CRT utilization strategy that does not delay implantation to the post‐discharge period following HF hospitalization may be appropriate. Patients who require hospitalization for HF despite having CRT already in place had worse outcomes compared with patients with no CRT. This observation is likely explained by these patients representing a particularly high‐risk subgroup. Randomized trials should be aimed at identifying the appropriate timing of CRT implantation relative to hospitalization for HF, as well as identification of specific characteristics that may affect patients' ability to derive the intended benefits of CRT.

## CONFLICTS OF INTEREST

The authors declare no potential conflict of interests.

## Supporting information


**TABLE S1** Mortality by CRT implant group comparing results of sensitivity analysis to primary analysis for the similar time period*

**TABLE S2** Heart failure hospitalization by CRT implant group comparing results of sensitivity analysis to primary analysis for the similar time period*

## References

[1] Benjamin EJ , Blaha MJ , Chiuve SE , et al. Heart disease and stroke Statistics‐2017 update: a report from the American Heart Association. Circulation. 2017;135:e146‐e603. 28122885 10.1161/CIR.0000000000000485 PMC5408160

[2] Mozaffarian D , Benjamin EJ , Go AS , et al. Heart disease and stroke Statistics‐2016 update: a report from the American Heart Association. Circulation. 2016;133:e38‐e360. 26673558 10.1161/CIR.0000000000000350

[3] Cleland JGF , Calvert MJ , Verboven Y , Freemantle N . Effects of cardiac resynchronization therapy on long‐term quality of life: an analysis from the Cardiac resynchronisation‐heart failure (CARE‐HF) study. Am Heart J. 2009;157:457‐466. 19249415 10.1016/j.ahj.2008.11.006

[4] Cleland JG , Daubert JC , Erdmann E , et al. The effect of cardiac resynchronization on morbidity and mortality in heart failure. N Engl J Med. 2005;352:1539‐1549. 15753115 10.1056/NEJMoa050496

[5] McAlister FA , Ezekowitz J , Hooton N , et al. Cardiac resynchronization therapy for patients with left ventricular systolic dysfunction: a systematic review. JAMA. 2007;297:2502‐2514. 17565085 10.1001/jama.297.22.2502

[6] Khazanie P , Hammill BG , Qualls LG , et al. Clinical effectiveness of cardiac resynchronization therapy versus medical therapy alone among patients with heart failure: analysis of the ICD registry and ADHERE. Circ Heart Fail. 2014;7:926‐934. 25227768 10.1161/CIRCHEARTFAILURE.113.000838 PMC4244212

[7] Epstein AE , Dimarco JP , Ellenbogen KA , et al. AHA/HRS 2008 guidelines for device‐based therapy of cardiac rhythm abnormalities: a report of the American College of Cardiology/American Heart Association task force on practice guidelines (writing committee to revise the ACC/AHA/NASPE 2002 guideline update for implantation of cardiac pacemakers and Antiarrhythmia devices) developed in collaboration with the American Association for Thoracic Surgery and Society of Thoracic Surgeons. J Am Coll Cardiol. 2008;51:e1‐e62. 18498951 10.1016/j.jacc.2008.02.032

[8] Tracy CM , Epstein AE , Darbar D , et al. 2012 ACCF/AHA/HRS focused update of the 2008 guidelines for device‐based therapy of cardiac rhythm abnormalities: a report of the American College of Cardiology Foundation/American Heart Association task force on practice guidelines. J Thorac Cardiovasc Surg. 2012;144:e127‐e145. 23140976 10.1016/j.jtcvs.2012.08.032

[9] Moss AJ , Hall WJ , Cannom DS , et al. Cardiac‐resynchronization therapy for the prevention of heart‐failure events. N Engl J Med. 2009;361:1329‐1338. 19723701 10.1056/NEJMoa0906431

[10] Bristow MR , Saxon LA , Boehmer J , et al. Cardiac‐resynchronization therapy with or without an implantable defibrillator in advanced chronic heart failure. N Engl J Med. 2004;350:2140‐2150. 15152059 10.1056/NEJMoa032423

[11] Pokorney SD , Al‐Khatib SM , Sun JL , et al. Sudden cardiac death after acute heart failure hospital admission: insights from ASCEND‐HF. Eur J Heart Fail. 2018;20:525‐532. 29266564 10.1002/ejhf.1078

[12] Smaha LA . The American Heart Association get with the guidelines program. Am Heart J. 2004;148:S46‐S48. 15514634 10.1016/j.ahj.2004.09.015

[13] Piccini JP , Hernandez AF , Dai D , et al. Use of cardiac resynchronization therapy in patients hospitalized with heart failure. Circulation. 2008;118:926‐933. 18697821 10.1161/CIRCULATIONAHA.108.773838

[14] Labresh KA , Ellrodt AG , Gliklich R , Liljestrand J , Peto R . Get with the guidelines for cardiovascular secondary prevention: pilot results. Arch Intern Med. 2004;164:203‐209. 14744845 10.1001/archinte.164.2.203

[15] Curtis LH , Greiner MA , Hammill BG , et al. Representativeness of a national heart failure quality‐of‐care registry: comparison of OPTIMIZE‐HF and non‐OPTIMIZE‐HF Medicare patients. Circ Cardiovasc Qual Outcomes. 2009;2:377‐384. 20031864 10.1161/CIRCOUTCOMES.108.822692 PMC2801895

[16] Heidenreich PA , Hernandez AF , Yancy CW , Liang L , Peterson ED , Fonarow GC . Get with the guidelines program participation, process of care, and outcome for Medicare patients hospitalized with heart failure. Circ Cardiovasc Qual Outcomes. 2012;5:37‐43. 22235067 10.1161/CIRCOUTCOMES.110.959122

