# Supplementary material for: Timing of cardiac resynchronization therapy device implantation in heart failure patients and its association with outcomes
Source: Clin Cardiol. 2018 Dec 26;42(2):256–63. doi: 10.1002/clc.23135 (PMC6386168; doi:10.1002/clc.23135)
Supplement: Supplementary file 2 — TABLE S2 Heart failure hospitalization by CRT implant group comparing results of sensitivity analysis to primary analysis for the similar time period* [file CLC-42-256-s001.docx]

**Supplemental Table 2.** Heart failure hospitalization by CRT implant group comparing results of sensitivity analysis to primary analysis for the similar time period*

|  | **Heart failure hospitalization** | | |
| --- | --- | --- | --- |
|  | **Sensitivity** | **Original** | |
|  | *2010-2012* | *2008-10* | *2011-12* |
| *CRT in place at admission* |  |  |  |
| N | 848 | 844 | 738 |
| Total events | 368 | 501 | 293 |
| Event rate at 1 year (95% CI‡) | 48.2% (44.5,52.1) | 46.1% (42.8,49.6) | 46.0% (42.0,50.4) |
| HR (95% CI) CRT vs. no CRT | 1.20 (1.04, 1.39) | 1.18 (1.06, 1.30) | 1.27 (1.10, 1.46) |
| P | 0.010 | 0.0021 | 0.0012 |
| *CRT implanted during hosp* |  |  |  |
| N | 462 | 624 | 324 |
| Total events | 107 | 207 | 63 |
| Event rate at 1 year | 20.3% (16.7,24.5) | 22.1% (19.0,25.6) | 18.6% (14.6,23.8) |
| HR (95% CI) CRT vs. no CRT | 0.48 (0.38, 0.59) | 0.59 (0.51, 0.69) | 0.50 (0.39, 0.65) |
| P | <.0001 | <.0001 | <.0001 |
| *CRT prescribed at discharge* |  |  |  |
| N | 398 | 266 | 377 |
| Total events | 140 | 133 | 116 |
| Event rate at 1 year | 38.5% (33.4,44.5) | 38.5% (33.0,44.8) | 36.9% (31.5,43.2) |
| HR (95% CI) CRT vs. no CRT | 1.04 (0.86, 1.26) | 0.98 (0.82, 1.18) | 1.03 (0.85, 1.26) |
| P | 0.67 | 0.86 | 0.75 |
| *No CRT* |  |  |  |
| N | 1,696 | 3,836 | 3,081 |
| Total events | 621 | 1906 | 987 |
| Event rate at 1 year | 38.1% (35.6,40.8) | 38.8% (37.3,40.4) | 36.5% (34.6,38.5) |

* Event rates are Kaplan-Meier rates. All P-values and hazard ratio are from adjusted Fine and Gray models. Follow-up duration shown as median (IOR). Models contain the following covariates: age, gender, race, left ventricular ejection fraction, systolic blood pressure, heart rate, body mass index, medical history (anemia, prior atrial arrhythmia, prior cerebrovascular accident or transient ischemic attack, ischemic heart disease, pulmonary disease, depression, diabetes, hypertension, hyperlipidemia, peripheral artery disease, renal insufficiency, chronic dialysis, smoking, prior revascularization), medications at discharge (angiotensin converting enzyme-inhibitor or angiotensin II receptor blocker, beta blocker, aldosterone antagonist, anticoagulant), and hospital characteristics (geographic region, rural location, teaching hospital, number of beds, and whether the hospital performs heart transplants). Missing values for covariates were imputed using multiple imputation (25 iterations).

CI = confidence interval

CRT = cardiac resynchronization therapy

HR = hazard ratio
